# Complete coding sequence of goose-derived cluster 3.2 Tembusu virus from Anhui province of China in October 2024

**DOI:** 10.1128/mra.00431-25

**Published:** 2025-07-07

**Authors:** Lei Zhao, Jie Zhu, Shao-Jun He, Peng-Fei Ye, Jie Kong, Liu-Jun Zhang

**Affiliations:** 1 Anhui Key Laboratory of Poultry Infectious Disease Prevention and Control, Anhui Science and Technology University 177515, Fengyang City, Anhui, China; 2 Anhui Engineering Technology Research Center of Pork Quality Control and Enhance, Anhui Science and Technology University 177515, Fengyang City, Anhui, China; Queens College Department of Biology, Queens, New York, USA

**Keywords:** Tembusu virus, coding sequence, China

## Abstract

In October 2024, a novel circulating Tembusu virus (TMUV) strain was isolated and characterized from an out-of-season breeding goose with pancreatic necrosis from Anhui province of China. Phylogenetic analysis revealed that the AHWH isolate shared the highest nucleotide identity with goose-derived TMUV/Goose/CHN/2022/HQ-22 and was classified into TMUV Cluster 3.2.

## ANNOUNCEMENT

Tembusu virus (TMUV) belongs to the genus *Orthoflavivirus* in the family *Flaviviridae*. Since 2010, TMUV has caused a severe disease characterized by a drop in egg production and neurological signs in poultry in China (1–4). Here, we report the complete coding sequence of TMUV strain AHWH isolated from an out-of-season breeding goose located in Wuhe County (33.1465°N, 117.9923°E), Anhui Province, China in October 2024.

The animal study was reviewed and approved by Anhui Science and Technology University Committee of Animal Experiments approval ID: 2025076. The AHWH strain, isolated from a 44-day-old goose that died of pancreatic necrosis and tested TMUV-positive by reverse transcription-polymerase chain reaction (RT-PCR), was recovered by standard allantoic cavity inoculation of 10-day-old specific-pathogen-free (SPF) chicken embryos (5). Viral RNA was extracted from the allantoic fluid using the MiniBEST Universal RNA Extraction Kit (TaKaRa, Beijing, China). Library preparation and Illumina sequencing were performed at Shanghai Tanpu Biotechnology Co., Ltd. (Shanghai, China). Briefly, the NEBNext Ultra II RNA Library Prep Kit (New England Biolabs, Ipswich, MA, USA) was used to construct paired-end libraries. After adapter ligation, 10 cycles of PCR amplification were performed to enrich the sequencing target. Sequencing was performed on an Illumina NovaSeq 6000 System (Illumina, San Diego, CA, USA) to generate 150 bp paired-end reads. Raw reads were processed with Fastp (6) to remove adapters and low-quality bases (Phred score <20). Host-derived reads (including ribosomal RNAs) were removed using BBMAP v38.51 (7). *De novo* genome assembly was performed using SPAdes v3.14.1 (8). These extracted assembled scaffolds limited the minimum contig length to 100 bases, with the best BLAST hits to the National Center of Biotechnology Information (NCBI) nucleotide database. A total of 6,928,796 raw reads were obtained. The final consensus sequence was generated from 552,647 filtered reads with an average depth of 4.86 × 10^3^. Taxonomic identification confirmed it as TMUV through nucleotide BLAST webserver (https://blast.ncbi.nlm.nih.gov/Blast.cgi). Subsequently, the Open Reading Frame (ORF) of this strain was aligned by the MUSCLE program in MEGA version 12 together with other selected TMUV strains from GenBank (9). A neighbor-joining phylogeny was reconstructed using the Maximum Composite Likelihood substitution model in MEGA12 with the bootstrap test (1,000 replicates). Default parameters were used for all software unless otherwise specified.

**Fig 1:**
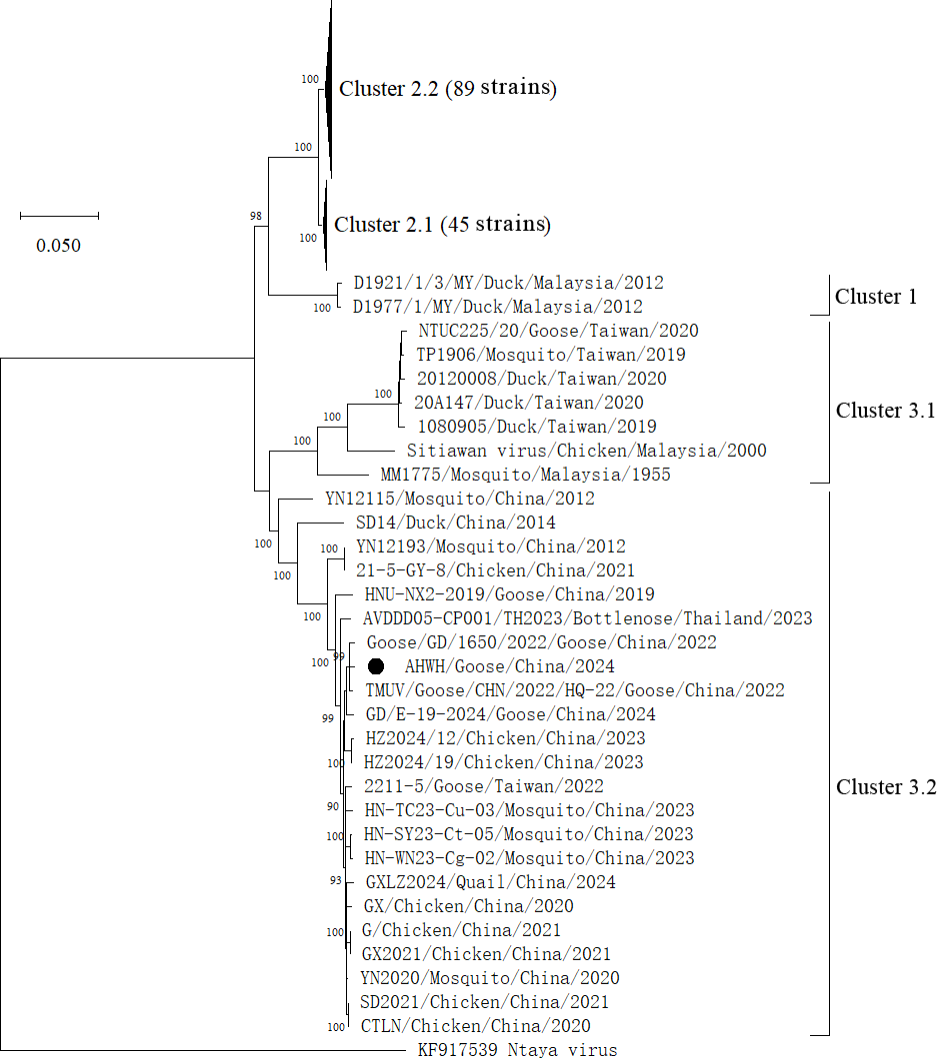
Neighbor-joining phylogeny of TMUV based on the ORF nucleotide sequences. Reference sequences were retrieved from GenBank. Multiple sequence alignment was performed using the MUSCLE program, and the Neighbor-joining phylogenetic tree was reconstructed using the Maximum Composite Likelihood substitution model with the bootstrap test (1,000 replicates) in MEGA version 12. Only bootstrap values exceeding 80% are displayed at branch nodes. Virus strains are annotated with strain name, host, country, and year of isolation. The TMUV AHWH isolate is denoted by a solid circle. The Ntaya isolate was used as the outgroup.

The final consensus sequence of the AHWH isolate was 11,030 bp in length with 49.18% GC content and contained a unique ORF of 10,278 bp encoding a polyprotein of 3,425 amino acids, along with a 79 bp 5′ untranslated region (UTR) and a 673 bp 3′ UTR. Compared to the TMUV/Goose/CHN/2022/HQ-22 strain (OR909676), our sequence lacked a 15 bp sequence in the 5′ UTR but had an additional 50 bp sequence in the 3′ UTR. The ORF sequence alignments revealed that the AHWH strain shared 85.27%–99.35% nucleotide identities with the published TMUV strains. Specifically, it showed the highest similarity to the goose-derived strain TMUV/Goose/CHN/2022/HQ-22 (99.35%). Phylogenetic analyses of the ORF indicated that the AHWH isolate was grouped into Cluster 3.2 (Fig. 1). Of note, the out-of-season goose farming period in Southeast China overlapped with the local mosquito activity season, thereby likely facilitating interspecies transmission of Cluster 3.2 TMUV between geese and mosquitoes (10–13).

## Data Availability

Raw reads were deposited in SRA under accession number PRJNA1255028. The assembled genomic sequence of the TMUV AHWH isolate has been deposited in GenBank under accession number PV541690.

## References

[1] Su J, Li S, Hu X, Yu X, Wang Y, Liu P, Lu X, Zhang G, Hu X, Liu D, Li X, Su W, Lu H, Mok NS, Wang P, Wang M, Tian K, Gao GF. 2011. Duck egg-drop syndrome caused by BYD virus, a new Tembusu-related flavivirus. PLoS One 6:e18106. doi:10.1371/journal.pone.0018106 21455312 PMC3063797

[2] Zhu Y, Hu Z, Lv X, Huang R, Gu X, Zhang C, Zhang M, Wei J, Wu Q, Li J, Zhang R, Cao S, Yin D, Wang B, Liu G, Wang G. 2022. A novel Tembusu virus isolated from goslings in China form a new subgenotype 2.1.1. Transbound Emerg Dis 69:1782–1793. doi:10.1111/tbed.14155 33993639

[3] Yu Z, Ren H, Sun M, Xie W, Sun S, Liang N, Wang H, Ying X, Sun Y, Wang Y, Zheng Y, Hu X, Su J. 2022. Tembusu virus infection in laying chickens: Evidence for a distinct genetic cluster with significant antigenic variation. Transbound Emerg Dis 69:e1130–e1141. doi:10.1111/tbed.14402 34821052

[4] Yan D, Li X, Wang Z, Liu X, Dong X, Fu R, Su X, Xu B, Teng Q, Yuan C, Zhang Z, Liu Q, Li Z. 2022. The emergence of a disease caused by a mosquito origin cluster 3.2 Tembusu virus in chickens in China. Vet Microbiol 272:109500. doi:10.1016/j.vetmic.2022.109500 35792374

[5] WOAH. 2023. Chapter 3.3.4: Avian influenza (infection with avian influenza viruses). In Manual of diagnostic tests and vaccines for terrestrial animals

[6] Chen S. 2023. Ultrafast one-pass FASTQ data preprocessing, quality control, and deduplication using fastp. Imeta 2:e107. doi:10.1002/imt2.107 38868435 PMC10989850

[7] Bushnell B. 2018. Bbtools: a suite of fast, multithreaded bioinformatics tools designed for analysis of DNA and RNA sequence data. https://jgi.doe.gov/data-and-tools/bbtools.

[8] Prjibelski A, Antipov D, Meleshko D, Lapidus A, Korobeynikov A. 2020. Using SPAdes de novo assembler. Curr Protoc Bioinformatics 70:e102. doi:10.1002/cpbi.102 32559359

[9] Kumar S, Stecher G, Suleski M, Sanderford M, Sharma S, Tamura K. 2024. MEGA12: molecular evolutionary genetic analysis version 12 for adaptive and green computing. Mol Biol Evol 41:msae263. doi:10.1093/molbev/msae263 39708372 PMC11683415

[10] Hu D, Wu C, Wang R, Yao X, Nie K, Lv Q, Fu S, Yin Q, Su W, Li F, Xu S, He Y, Liang G, Li X, Wang H. 2023. Persistence of Tembusu Virus in Culex tritaeniorhynchus in Yunnan province, China. Pathogens 12:490. doi:10.3390/pathogens12030490 36986412 PMC10058924

[11] Wu Q, Sun D, Zaman W, Wang F, Huang D, Ma H, Wang S, Liu Y, Liu P, Zeng X, Yuan Z, Xia H. 2024. Detection and evolutionary characterization of arboviruses in mosquitoes and biting midges on Hainan Island, China, 2019-2023. PLoS Negl Trop Dis 18:e0012642. doi:10.1371/journal.pntd.0012642 39480881 PMC11556698

[12] Yang Q, Ding Y, Yao W, Chen S, Jiang Y, Yang L, Bao G, Yang K, Fan S, Du Q, Wang Q, Wang G. 2023. Pathogenicity and interspecies transmission of cluster 3 Tembusu virus strain TMUV HQ-22 isolated from geese. Viruses 15:2449. doi:10.3390/v15122449 38140690 PMC10747935

[13] Wang X, Chen X, Ding Y, Xu P, Wang C, Wei X, Peng H, Cui C, Kang O, Chen Y, Wei Z, Huang W, Qin Y. 2025. Isolation, identification, and pathogenicity evaluation of a novel Cluster 3 Tembusu virus isolated from geese in China. Poult Sci 104:104684. doi:10.1016/j.psj.2024.104684 39718055 PMC11733047

